# Buccolingual and arch distribution patterns of permanent teeth impactions in an Egyptian population: a CBCT-based retrospective analysis

**DOI:** 10.1186/s12903-025-06955-7

**Published:** 2025-10-02

**Authors:** Farah Y. Eid, Ahmed R. Elkalza, Ahmed M. Madian, Yomna M. Yacout

**Affiliations:** Department of Orthodontics, Faculty of Dentistry, Alexandria University, Champollion St, P. O. Box: 21521, Azarita, Alexandria Egypt

**Keywords:** Impaction, Cone beam computed tomography, Prevalence, Diagnostic imaging, Dental radiography

## Abstract

**Background:**

The prevalence of impacted teeth fluctuates across various ethnicities and geographical regions. Therefore, the aim of the study was to investigate the prevalence of impacted permanent teeth, excluding third molars, in the Egyptian population, and to report, using cone beam computed tomography (CBCT) scans, the characteristics of the impacted teeth.

**Materials and methods:**

A retrospective study design was employed to address the study aim, where 4522 pre-treatment panoramic radiographs were collected from several radiographic centers in Alexandria, Egypt. The presence of any impacted permanent tooth, except third molars, was documented based on the stage of root development, and the patient’s CBCT scan was retrieved from the patient’s file for evaluation of the arch distribution (maxillary or mandibular), the side distribution (right or left), and the buccolingual position of each impacted tooth (labial/buccal, lingual/palatal, or mid-ridge). Frequencies and percentages were calculated for all variables.

**Results:**

The results showed that 315 subjects (7%) presented with impactions, with 191 subjects (60.6%) exhibiting a single impacted tooth, and three subjects (0.9%) exhibiting six or more impacted teeth. Canines constituted 72.1% of the impactions, followed by second premolars (16%). A significantly larger number of central incisors, lateral incisors, canines and first premolars were impacted in the maxillary arch than in the mandibular arch (*p* < 0.001). A significantly larger number of second premolars was impacted in the mandibular arch than the maxillary arch (*p* < 0.001). No significant side differences were found for all tooth categories (*p* = 0.63). Most of the impacted maxillary central incisors, canines, and premolars, and the impacted mandibular canines and second premolars were located labially. All impacted mandibular first premolars were located labially.

**Conclusions:**

The prevalence of impactions is 7% in the non-syndromic Egyptian population. Patients present most commonly with a single impacted tooth. The higher prevalence of impactions in the maxillary arch and on the labial side of the arch warrants thorough clinical examination and radiographic assessment of these areas. Clinicians should pay special attention to canine impaction.

## Introduction

Tooth impaction is a problem commonly encountered by orthodontists. One of the reasons patients seek orthodontic treatment when teeth fail to erupt is to improve smile esthetics. However, tooth impaction may have other detrimental consequences on the patient’s oral health. One consequence is that spaces remain in the dental arch when a deciduous tooth is lost and its permanent successor fails to erupt. The patient’s mastication and speech may be negatively affected by the space that ensues. Moreover, the normal occlusal development and eruption sequence of the neighboring teeth may be disturbed [1]. Root resorption and loss of vitality of the adjacent teeth may also take place [2, 3]. Early diagnosis of tooth impactions allows for timely management, hence preventing more prolonged and costly interventions later in life [4]. 

A tooth is defined as impacted when it fails to erupt because of its displacement or due to a mechanical obstruction [5]. A local mechanical obstruction may be a retained deciduous tooth, a supernumerary tooth, or a local pathology such as an odontoma or a cyst that blocks the eruption path of permanent teeth [4, 6]. Impactions may also result from trauma to the deciduous predecessor, which deflects the permanent tooth from its normal eruption pathway [7]. In addition, tooth impactions may be related to genetic predispositions [8], and/or systemic and syndromic conditions [9]. On another note, the prevalence of impacted teeth shows considerable diversity in different world regions, as well as in their distribution among the maxillary and mandibular arches [10–12]. 

A patient may present with a single impacted tooth or multiple impacted teeth in the maxilla, mandible, or both. Previous research has demonstrated that the presence of an impacted maxillary incisor may have a role in impaction of the ipsilateral canine [13]. The presence of multiple impacted teeth and their position relative to each other may possibly influence the orthodontic treatment decision. An impacted tooth may be located labial/buccal, lingual/palatal, or in the middle of the alveolar ridge. The location of the impaction relative to other teeth in the dental arch may affect the orthodontic treatment plan and the surgical procedures employed to expose and guide the impacted tooth into place [1, 14]. 

The presence of impacted teeth can be verified by examining panoramic radiographs [1]. However, to specify the position of the tooth impaction, further investigations are mandatory. Periapical radiographs can be used with the parallax technique to identify the buccolingual position of the tooth [14]. Also, occlusal radiographs can be utilized for localization of the impacted tooth [1]. Currently, the use of cone beam computed tomography (CBCT) offers the advantage of simplifying the localization of impacted teeth with better accuracy [15]. 

Although the prevalence of impacted maxillary canines in the Egyptian population has been reported previously [16], the buccolingual location of the impaction was not specified. Understanding the variation in the buccolingual locations is important for clinical decision-making, especially regarding interceptive orthodontic treatment and surgical procedures to either expose or extract the impacted teeth [14]. Additionally, the prevalence of impacted incisors, premolars and molars in the Egyptian population has not been documented in former research. Therefore, the objective of the current study was to investigate the prevalence of impacted incisors, canines, and premolars in a sample of the Egyptian population, and to report, using CBCT scans, the characteristics of the impacted teeth with regards to the arch distribution (maxillary or mandibular), side distribution (right or left), and buccolingual position of the impaction (labial/buccal, lingual/palatal, or mid-ridge).

## Materials and methods

A retrospective radiographic study design was used to address the aim of the study. Ethical approval was obtained from the Institutional Review Board of the Faculty of Dentistry, Alexandria University (IORG:0008839, Protocol no. 0906–04/2024) before starting the study.

### Study setting and subjects

Pre-treatment radiographs of dental patients were collected from Alexandria University’s dental radiographic center and several private radiographic centers in Alexandria, Egypt. The inclusion criteria included the availability of good quality panoramic radiographs, and in turn, CBCT scans for those diagnosed with an impacted incisor, canine, premolar, or molar. Conversely, radiographs of cleft lip and/or palate patients or syndromic patients were excluded.

### Sample size calculation

The sample size was based on a 95% confidence level, 5% margin of error, and an estimated prevalence of permanent teeth impaction ranging from 9.1% to 44.1% as reported in previous studies [12, 17–19]. The minimum required sample size was calculated to be 379 radiographs, increased to 455 to make up for procedural problems [20]. Sample size estimation was performed using an online Sample size calculator [21]. 

### Records’ screening

All the panoramic radiographs obtained from the different radiographic centers were screened by one investigator (A. E.) and were assessed for conformity with the aforementioned eligibility criteria. The presence of any impacted incisor, canine, premolar, or molar was documented based on findings from the panoramic radiographs. A tooth was defined as impacted if it was unerupted despite its root showing more than three-quarters of its anticipated final length [1]. Additionally, a tooth was considered impacted if there was any physical obstruction in its path of eruption [1]. In case of the identification of an impaction in the panoramic radiograph, the type of impacted tooth was recorded, and the CBCT scan of the patient was retrieved from the patient’s file. CBCT scans were imported in DICOM format into OnDemand3D™ software (Cybermed Inc., Seoul, Korea) for further analysis.

### Outcomes

The retrieved CBCT scans were examined by one investigator (A. E.) to confirm the initial diagnosis of tooth impaction. The three-dimensional volume rendering was first assessed to determine the arch distribution (maxillary or mandibular) and the side distribution (right or left) of the impacted tooth. Afterwards, sequential axial cuts through the area of each impacted tooth were evaluated to record the buccolingual position of the impacted tooth based on the location of the impacted tooth’s crown relative to the neighboring teeth (labial/buccal, lingual/palatal, or mid-ridge) [2]. 

### Reliability

A subset of 50 radiographs was re-evaluated after a two-week interval by the same examiner, and the Cohen’s kappa coefficient was calculated to assess intra-examiner agreement. The kappa value was 0.88, indicating excellent agreement [22]. 

### Statistical analysis

Frequencies and percentages were calculated for all variables. For key proportions such as impaction prevalence and subgroup distributions, 95% confidence intervals (CIs) were calculated using Wilson’s method to provide more precise estimates. Group comparisons (e.g., by arch, side, location, and tooth type) were conducted using the Chi-square test with Monte Carlo correction when appropriate. To assess the strength of associations, effect sizes were calculated using Cramér’s V and interpreted as follows: values < 0.1 indicate a negligible association, 0.1–0.3 a small association, 0.3–0.5 a moderate association, and > 0.5 a strong association [23]. Significance level was set at p-value < 0.05. Data was analyzed using IBM SPSS for Windows (Version 26.0).

## Results

A total of 4522 panoramic radiographs were collected from 10 different radiographic centers, substantially exceeding the minimum sample size requirement, thus improving the study’s statistical power and generalizability. The characteristics of the studied sample are summarized in Table 1. The majority of the subjects were females (*n* = 3096 (65.9%)). The prevalence of impactions in the sample studied was 7% (95% CI = 6.2% to 7.8%). The mean age of the patients who had impacted teeth was 17.06 ± 5.29 years. Out of the 315 patients with impactions, 113 were males and 202 were females. Most patients (60.6%) had one impacted tooth, while less than 1% of the patients had six or more impacted teeth.

**Table 1:** Sample characteristics

Characteristics of the study sample (*N* = 4522 participants, 4700 teeth)		
Gender, *n* (%)		
Male		1426 (30.3%)
Female		3096 (65.9%)
Age, years		
Mean (SD)		16.75 (5.26)
Minimum-Maximum		8.00–42.00
Impaction prevalence, *n* participants (%)		
* n* (%)		315 (7%)
95% CI		6.2% − 7.8%
Characteristics of the participants with impacted teeth(***N*** = 315 participants)		
Gender,*n* (%)		
Male		113 (35.9%)
Female		202 (64.1%)
Age, years		
Mean (SD)		17.06 (5.29)
Minimum-Maximum		9.00–40.00
Number of impacted teeth per person		
1 tooth	*n* (%)	191 (60.6%)
	95% CI	55.1% − 65.9%
2 teeth	*n* (%)	94 (29.8%)
	95% CI	25.1% − 35.1%
3 teeth	*n* (%)	20 (6.3%)
	95% CI	4.1% − 9.6%
4 teeth	*n* (%)	7 (2.2%)
	95% CI	1.1% − 4.5%
6 teeth	*n* (%)	1 (0.3%)
	95% CI	0.1% − 1.8%
8 teeth	*n* (%)	1 (0.3%)
	95% CI	0.1% − 1.8%
12 teeth	*n* (%)	1 (0.3%)
	95% CI	0.1% − 1.8%

Table 2 shows the characteristics of the impacted teeth in the study sample. A total of 494 impacted teeth were reported. The canines comprised the majority of impacted teeth (72.1%), followed by second premolars (16%) and central incisors (7.3%). Only one impacted molar was reported in the investigated sample. A significantly greater number of teeth were impacted in the maxillary arch (77.1%, 95% CI: 73.2%–80.6%) than in the mandibular arch (22.9%, 95% CI: 19.4%–26.8%) (*p* < 0.001). The strength of this association was moderate (Cramér’s V = 0.39). The distribution of impacted teeth was 52.4% (95% CI: 48.0%–56.8%) on the left side and 47.6% (95% CI: 43.2%–52.0%) on the right side, and this association was negligible, with a Cramér’s V = 0.086. Most impactions were labial at 64% (95% CI: 59.6%–68.1%), followed by lingual at 24.5% (95% CI: 20.9%–28.5%) and mid-ridge at 11.5% (95% CI: 9.0%–14.7%).

**Table 2:** Characteristics of the impacted teeth in the study sample (*N* = 494 teeth)

	*n* (%)	95% CI
Type of impacted tooth		
Central incisor	36 (7.3%)	5.3% − 9.9%
Lateral incisor	10 (2%)	1.1% − 3.7%
Canine	356 (72.1%)	67.9% − 75.8%
First premolar	12 (2.4%)	1.4% − 4.2%
Second premolar	79 (16%)	13% − 19.5%
First molar	1 (0.2%)	0% − 1.1%
Arch of impacted tooth		
Maxillary	381 (77.1%)	73.2% − 80.6%
Mandibular	113 (22.9%)	19.4% − 26.8%
Side of impacted tooth		
Left side	259 (52.4%)	48% − 56.8%
Right side	235 (47.6%)	43.2% − 52%
Location of impacted tooth		
Labial	316 (64%)	59.6% − 68.1%
Lingual	121 (24.5%)	20.9% − 28.5%
Mid-ridge	57 (11.5%)	9% − 14.7%

A significantly larger number of central incisors, lateral incisors, canines and first premolars were impacted in the maxillary arch than in the mandibular arch as shown in Table 3. On the other hand, a significantly larger number of second premolars was impacted in the mandibular arch than the maxillary arch. The impacted first molar was located in the mandibular arch. There was no significant difference in impaction distribution between the right and left sides for all the tooth categories (Table 4).

**Table 3:** Distribution of impacted teeth according to arch

Type of impacted tooth	Maxillary (*n* = 381)		Mandibular (*n* = 113)		*P* value
	*N* (%)	95% CI	*N* (%)	95% CI	
Central incisor	36 (9.4%)	6.9% − 12.8%	0 (0%)	0% − 3.3%	< 0.001*
Lateral incisor	8 (2.1%)	1.1% − 4.1%	2 (1.8%)	0.5% − 6.2%	
Canine	296 (77.7%)	73.2% − 81.6%	60 (53.1%)	43.9% − 62%	
First premolar	7 (1.8%)	0.9% − 3.7%	5 (4.4%)	1.9% − 9.9%	
Second premolar	34 (8.9%)	6.5% − 12.2%	45 (39.8%)	31.1% − 49%	
First molar	0 (0%)	0% − 1%	1 (0.9%)	0.2% − 4.8%	

Chi-square test with Monte Carlo correction was used

*statistically significant at *p*-value < 0.05

**Table 4:** Distribution of impacted teeth according to side

Type of impacted tooth	Left side (*n* = 259)		Right side (*n* = 235)		*P* value
	*N* (%)	95% CI	*N* (%)	95% CI	
Central incisor	22 (8.5%)	5.7% − 12.5%	14 (6%)	3.6% − 9.8%	0.63
Lateral incisor	7 (2.7%)	1.3% − 5.5%	3 (1.3%)	0.4% − 3.7%	
Canine	184 (71%)	65.2% − 76.2%	172 (73.2%)	67.2% − 78.4%	
First premolar	6 (2.3%)	1.1% − 5%	6 (2.6%)	1.2% − 5.5%	
Second premolar	40 (15.4%)	11.6% − 20.3%	39 (16.6%)	12.4% − 21.9%	
First molar	0 (0%)	0% − 1.5%	1 (0.4%)	0.1% − 2.4%	

Chi-square test with Monte Carlo correction was used

*statistically significant at *p*-value < 0.05

Regarding the location of the impactions in the maxillary arch, most of the impacted central incisors, canines, first premolars and second premolars were located labially (88.9%, 54.7%, 71.4, and 58.85%, respectively) as shown in Table 5. Maxillary lateral incisors were equally impacted on the labial and palatal sides, while central incisors and premolars were equally impacted on the palatal side and mid-ridge. Regarding the location of the impactions in the mandibular arch, the majority of canines and second premolars were labially impacted (96.7% and 64.5%, respectively). All impacted mandibular first premolars were labially impacted, and the impacted mandibular first molar was located mid-ridge.

**Table 5:** Distribution of impacted teeth according to location in the arch

Type of impacted tooth		Maxillary arch					Mandibular arch				
		Labial	Palatal	Mid-ridge	Total	*p*-value	Labial	Lingual	Mid-ridge	Total	*p*-value
Central incisor	*N* (%)	32 (88.9%)	2 (5.55%)	2 (5.55%)	36	< 0.001*	0 (0%)	0 (0%)	0 (0%)	0	< 0.001*
	95% CI	74.7% − 95.6%	1.5% − 18.1%	1.5% − 18.1%			0% − 1%	0% − 1%	0% − 1%		
Lateral incisor	*N* (%)	4 (50%)	4 (50%)	0 (0%)	8		1 (50%)	1 (50%)	0 (0%)	2	
	95% CI	21.5% − 78.5%	21.5% − 78.5%	0% − 32.4%			9.5% − 90.5%	9.5% − 90.5%	0% − 65.8%		
Canine	*N* (%)	162 (54.7%)	103 (34.8%)	31 (10.5%)	296		58 (96.7%)	2 (3.3%)	0 (0%)	60	
	95% CI	49% − 60.3%	29.6% − 40.4%	7.5% − 14.5%			88.6% − 99.1%	0.9% − 11.4%	0% − 6%		
First premolar	*N* (%)	5 (71.4%)	1 (14.3%)	1 (14.3%)	7		5 (100%)	0 (0%)	0 (0%)	5	
	95% CI	35.9% − 91.8%	2.6% − 51.3%	2.6% − 51.3%			56.6% − 100%	0% − 43.4%	0% − 43.4%		
Second premolar	*N* (%)	20 (58.8%)	7 (20.6%)	7 (20.6%)	34		29 (64.5%)	1 (2.2%)	15 (33.3%)	45	
	95% CI	42.2% − 73.6%	10.3% − 36.8%	10.3% − 36.8%			49.8% − 76.8%	0.4% − 11.6%	21.4% − 47.9%		
First molar	*N* (%)	0 (0%)	0 (0%)	0 (0%)	0		0 (0%)	0 (0%)	1 (100%)	1	
	95% CI	0% − 1%	0% − 1%	0% − 1%			0% − 79.3%	0% − 79.3%	20.7% − 100%		

Chi-square test with Monte Carlo correction was used

*statistically significant at *p*-value < 0.05

## Discussion

With dental impactions being a commonly encountered problem in the orthodontic practice, the present study aimed to investigate the prevalence of impacted incisors, canines, premolars, and molars in a sample of the Egyptian population. Additionally, the study aimed to describe the characteristics of the impacted teeth in terms of the arch distribution, the side distribution, and the buccolingual position, all of which have not been formerly reported by any Egyptian institutions or research articles. Third molars, on the other hand, were excluded in the current analysis. Recent research highlighted that unerupted third molars may be commonly extracted as a preventive measure [24]. However, this could not be ascertained in the current study owing to its retrospective nature, therefore third molars were excluded.

Female subjects comprised the majority of the study sample with a percentage of 69.5%. Previous research has shown that females generally have a more positive attitude towards oral health and that they often seek professional dental care more than males [25, 26]. This may have resulted in a larger number of female radiographic records being available for analysis, and accordingly, the higher impaction rate among females described in the present study (64.1%). This is in accordance with the impaction prevalence in a Greek population reported by Gisakis et al. [19], where impactions were more prevalent in females. Gender differences may also be attributed to the relatively smaller size of the skull, maxilla, and mandible in females than males [27], which may result in a tooth size-arch length discrepancy and subsequent impaction of the teeth due to lack of space.

In the study sample, the radiographs that showed impacted teeth were generally those of adolescents or young adults. A similar observation was recently reported by Choi et al. [28] where a higher prevalence of impactions was found in pediatric patients compared to adult patients. A possible reason for this observation is that patients often seek dental and/or orthodontic treatment at a young age when teeth fail to erupt in their expected timeframe. To ascertain whether a tooth was truly impacted or merely unerupted was based on the presence of a physical obstruction in its eruption route in addition to the stage of root development [1]. The presence of a non-erupted tooth with more than three-quarters of the root developed substantiated impaction, because the chance of a tooth to spontaneously erupt after completion of root formation is reduced [1]. 

Panoramic radiographs were initially employed in the screening phase, as they provide sufficient information about the teeth in both dental arches, as well as the surrounding structures [29]. They are also contemplated as an evaluation fundament in epidemiological investigations since they are both economic and practical [19]. However, for a more detailed evaluation of the identified impactions, CBCT scans were used as they offer three-dimensional multiplanar images, enabling the accurate diagnosis and the precise localization of impacted teeth [3, 30]. 

The prevalence of impaction in the current studied sample of Egyptians was 7%. Previous epidemiological studies reported a prevalence of impactions ranging between 2.94% and 44.1% [10, 12, 17–19, 28, 31–37]. The diversity in the prevalence of impacted teeth reported in the different studies could be accredited to the variability in the ethnic and genetic backgrounds, as well as the different eligibility criteria employed in the various studies, such as age and definition of impaction. In addition, Choi et al. [28] included cases that presented with systemic diseases characterized by multiple impacted teeth, such as cleidocranial dysostosis. Moreover, several studies included third molars in their assessment of impactions [18, 19, 32, 33, 35, 36]. To the best of our knowledge, no previous research reported the overall prevalence of impaction in an Egyptian population.

Approximately 60% of the patients with impactions in the sample studied had one impacted tooth only, whereas the occurrence of four or more impactions was rare. Similar results were previously reported by Fardi et al. [10], where 75.9% of the patients presented with one impacted tooth compared to 5.3% who presented with three or more impacted teeth. Likewise, Choi et al. [28] reported a prevalence of 68.2% for single tooth impactions compared to a prevalence of 1.7% for four or more tooth impactions. Multiple impactions are common in syndromic patients such as those suffering from cleidocranial dysplasia or cherubism, as well as in cleft patients [9], both of which were excluded in our study. The presence of multiple impactions of the permanent teeth in non-syndromic cases may result from traumatic dental injuries [38]. However, the occurrence of eruption disturbances following trauma to the primary incisors was previously shown to be relatively uncommon [7]. On the other hand, the presence of one impacted tooth or two bilaterally impacted teeth, especially bilaterally impacted canines, is more common [8]. 

Analysis of the records in the current study revealed that canines were the most impacted (72.1%), next were second premolars (16%), followed by central incisors (7.3%). The least impacted teeth were the molars, with only one tooth recorded in the sample studied, excluding third molars. Similarly, aside from third molars, canines were the most impacted teeth followed by premolars in Saudi [12], Indian [39], Greek [10], Turkish [17, 40], and Chinese [11] populations.

Further analysis of canine impactions revealed that they comprised 77.7% (296 teeth) of the total maxillary impactions, whereas mandibular canines contributed to 53.1% (60 teeth) of the mandibular impactions. The reported high impaction frequency of the maxillary canines relates to their long eruption path till reaching full occlusion [8]. Accordingly, they erupt later than the adjacent teeth and any lack of space in the maxillary dental arch may result in their impaction [5]. On the other hand, mandibular canine impaction is relatively infrequent, and its occurrence is uncommon in contrast to maxillary ones, which could be accredited to their early eruption in the mandibular arch, ahead of the first and second premolars [5]. 

As per the literature reports assessing the prevalence of premolar impactions, second premolars are more frequently impacted than first premolars, especially in the mandibular arch, followed by those in the maxillary arch [10, 12, 31, 32, 41]. These findings are in line with those reported in the present study, where a significantly larger number of second premolars were impacted in the mandibular arch than in the maxillary arch. This could be attributed to the sequence of eruption in the mandibular arch, where second premolars erupt last, preceded by the canines and the first premolars [5]. Accordingly, in cases with premature loss of deciduous teeth and potential space problems, the occurrence of second premolar impaction is more likely.

The impacted central and lateral incisors in the present study accounted for 7.3% and 2% of the total impactions, respectively, with the majority recorded in the maxillary arch. The absence or scarcity of impacted incisors in the mandibular arch was frequently documented in prior investigations [11, 12, 17, 19, 28, 42]. The rarity of mandibular incisor impaction may be attributed to its early eruption in the oral cavity thus evading obstacles to its path of eruption. On the other hand, the frequency of impacted maxillary incisors may be related to trauma to the anterior region which is common in children [38]. Moreover, the presence of supernumerary teeth may impede the eruption of maxillary incisors [43]. Previous research reported that most of the supernumerary teeth were found in the premaxilla [43, 44]. On another note, the high frequency of impacted maxillary central incisors in the studied population may have played a role in the high prevalence of impacted maxillary canines reported in the current study. Chaushu et al. [13] previously demonstrated a significant increase in the prevalence of impacted canines in case of the presence of an impacted central incisor on the same side.

Only one impacted mandibular first molar was recorded in the present study sample, which accounted for 0.2% of the total impactions. This aligns with molar impaction prevalences reported in former investigations, excluding third molars [11, 32, 42]. First molar impactions are not a common observation in panoramic radiographs [36]. A transient impaction of the first molar may occur against the distal surface of the deciduous second molar, however, the problem is usually managed at a young age [5]. Ectopic eruption of the first molar is one of the problems that the American Association of Orthodontists advises to watch for in 7-year-old children [45]. In such cases, if the orthodontist does not interfere early, the first molar may spontaneously erupt following loss of the deciduous second molar [5], with the resultant decrease in the arch length possibly causing impaction of other permanent teeth that erupt later on. Primary failure of eruption of the permanent first molars may also be encountered, nevertheless, it is rare [46]. Conversely, Choi et al. [28] reported a high prevalence of impacted first and second molars. The higher frequency of molar impactions in the aforementioned study may be attributed to the inclusion of cases with systemic diseases that are characterized by the presence of multiple impacted teeth including molars. Moreover, the records investigated in the abovementioned study were collected from multiple university dental hospitals, which may have overestimated the prevalence of molar impactions because of frequent referral of complex cases from the local dental clinics [28]. 

In the current study, comparable numbers of impacted teeth were reported on the right and left sides of both arches, with no significant differences for all tooth types. Analogous results were previously reported in several studies [17, 19, 31, 36]. Conversely, previous research on the prevalence of impacted canines shows that the majority of the existent literature demonstrate a predominance of impactions occurring on the left side compared to impactions on the right side [16, 42, 47, 48]. The disparity between the studies may be related to the multifactorial etiology of maxillary canine impaction [6]. 

Regarding the location of the impactions in the dental arch, the majority of the maxillary impactions, whether incisors, canines, or premolars were located on the labial side of the arch. According to the published literature, the frequency of palatal impactions is usually higher than that of buccal impactions, especially for maxillary canines [48–52], which contrasts with our reported findings. Contrarily, Kim et al. [2] reported three-times more buccal canine impactions than palatal impactions. The conflicting results may be attributed to the different ethnicities investigated in the different studies, with the possibility of population-specific variations. For example, the timing and sequence of eruption of the permanent teeth may vary in the different populations [53], thus resulting in different patterns of impaction. Furthermore, the inconsistency between the studies may have risen from differences in the imaging techniques and in the interpretation of the impacted teeth positions [29]. 

Additionally, Jacoby [54] highlighted the difficulty of confirming an exact rate for palatal versus buccal impactions, due to the multitude of local, genetic, and systemic factors involved in the etiology of each. With canine impactions for example, it was found that 85% of those located palatally had enough dental arch space allowing their eruption, in contrast to labially impacted canines, where only 17% of the cases presented with sufficient dental arch space [54]. Similarly, Yan et al. [55] reported that the main cause of buccal canine impaction was anterior transverse deficiency, whether dental or skeletal. In consequence, insufficient arch length is regarded as a primary etiologic factor in labial canine impaction [54, 55]. On the contrary, maxillary lateral incisor microdontia or agenesis were assumed to be the most common causes behind palatal canine impaction [54, 55], a finding that was not investigated in the present study.

Similarly, in the present study, more canine and premolar impactions were found on the labial side than on the lingual side in the mandibular arch. Analogous results were previously reported for mandibular canines [56], possibly because of the labial position of the bud during the tooth formation [57]. Moreover, the lack of space in the dental arch often results in displacement of the canine anteriorly and mesially where the symphysis is wider [56]. On the other hand, a search through the literature revealed that no previous research evaluated the location of premolar impactions.

From a clinical perspective, when planning orthodontic treatment, the location of an impacted tooth within the dental arch is of prime importance. The position of an impacted tooth, whether it is located on the labial/buccal, or the lingual/palatal side of the dental arch, or centered within the alveolar ridge, steers the treatment plan. Moreover, the spatial relationships between multiple impacted teeth dictate the specific surgical procedures needed to uncover the teeth and guide them into their proper positions [1, 14]. Furthermore, the results of the current study underline the importance of early screening using panoramic radiographs to allow timely detection and intervention of potential impactions. The role of panoramic radiographs in early prediction of maxillary canine impactions was highlighted in previous research [58]. 

### Limitations

Establishing the actual prevalence of tooth impaction in the population requires studying a representative random sample [10], which was not feasible due to ethical concerns regarding exposing healthy subjects to unnecessary radiation solely for research purposes. Moreover, the inclusion of cases referred to radiographic centers may have introduced selection bias because the individuals undergoing panoramic radiography are not a random sample of the general population. They are individuals with existing dental concerns and a higher likelihood of dental problems. Consequently, the reported prevalence of impactions may possibly be an over-estimation compared to the true prevalence in the broader population. Additionally, due to the retrospective nature of the research and the research setting, the dental history of the subjects under study was not readily available. Patients with a history of dental treatment that involved extraction of impacted teeth may have been included in the sample studied, thus underestimating the prevalence of impacted teeth.

Another limitation in the methodology of the study is that the radiography machines and the settings used in obtaining the radiographs were diverse due to the inclusion of different radiographic centers, which may have affected the quality of the radiographs and the subsequent analysis.

Furthermore, detailed statistical analysis of the characteristics of the impacted teeth within the different age and gender sub-groups was not undertaken in the current study, which may present a limitation when interpreting the clinical impact of the study findings. Previous research has demonstrated that the severity of angulation of impacted teeth was higher in older patients and in female patients [34]. Therefore, future research with a larger sample size that is specifically powered for sub-group comparisons is recommended to strengthen the clinical impact of the current study findings by providing deeper insights on how age and/or gender may influence the various characteristics of the impacted teeth.

In addition, a nationwide investigation should be conducted with a larger pool of patients from different regions of the country to expand the scope of the study.

## Conclusions

Within the limitations of the current study, it can be concluded that the prevalence of impactions is 7% in the non-syndromic Egyptian population, with patients presenting most commonly with a single impacted tooth. This highlights the importance of screening for individual impacted teeth to allow early detection and intervention. The higher prevalence of impactions in the maxillary arch and on the labial side of the arch warrants thorough clinical examination and CBCT radiographic assessment of these areas. Clinicians should pay special attention to canine impaction given that the most commonly impacted tooth is the maxillary canine, followed by the mandibular canine.

## Data Availability

The datasets used and/or analyzed during the current study are available from the corresponding author on reasonable request.
